# Experimental Comparison of Half-Value Layer Measurements Using a Solid-State X-ray Meter and an Ionization Chamber in General Radiography

**DOI:** 10.7759/cureus.117313

**Published:** 2026-10-01

**Authors:** Kunihiko Katagiri, Genta Ito, Toshiki Hirakado

**Affiliations:** 1 Department of Diagnostic Radiology, Faculty of Health and Welfare, Tokushima Bunri University, Takamatsu City, JPN; 2 Department of Radiology, Kochi University Hospital, Kochi, JPN; 3 Department of Diagnostic Imaging Technology and Clinical Support, Itsukaichi Memorial Hospital, Hiroshima, JPN

**Keywords:** cobia smart, diagnostic radiography, half-value layer, ionization chamber, measurement uncertainty, quality control, solid-state detector, x-ray dosimetry

## Abstract

Background: Half-value layer (HVL) is a fundamental descriptor of diagnostic X-ray beam quality. This study compared HVL measurements obtained with a solid-state X-ray meter (Cobia SMART, RTI Group, Mölndal, SWE) and an ionization-chamber dosimetry system across multiple tube-voltage and measurement-distance conditions.

Methods: Measurements were performed at 60, 80, 100, and 120 kV and source-to-detector distances (SDDs) of 100, 150, and 200 cm. The corresponding exposure settings were 50, 110, and 200 mAs, respectively. Three independent ionization-chamber HVL determinations and three Cobia readings were obtained for each condition. Agreement was evaluated using absolute and percentage differences and descriptive Bland-Altman analysis. A partial uncertainty assessment incorporated repeatability-based type A uncertainty and the 0.1 mm Al recording resolution of the Cobia data.

Results: The ionization-chamber coefficient of variation ranged from 0.09% to 2.38%, with a mean of 1.16%. Cobia showed identical recorded triplicate values at each tube voltage, with HVLs of 2.1, 2.9, 3.7, and 4.6 mm Al at 60, 80, 100, and 120 kV, respectively. Eleven of the 12 paired conditions differed by no more than 0.20 mm Al. The largest discrepancy occurred at 120 kV and 150 cm, where the difference was +0.466 mm Al (+11.27%). Bland-Altman analysis yielded a mean bias of +0.057 mm Al, with descriptive 95% limits of agreement from -0.240 to +0.353 mm Al.

Conclusions: Cobia SMART demonstrated stable recorded HVL values and generally close agreement with the ionization-chamber method across most tested conditions. However, a notable discrepancy was observed at 120 kV and 150 cm, indicating that agreement under one exposure condition should not be generalized across the entire diagnostic range. Cobia SMART may be a practical tool for rapid routine HVL quality-control measurements, with periodic comparison against a calibrated reference dosimetry system advisable after changes in measurement geometry or when unexpected HVL values are obtained.

## Introduction

Accurate characterization of X-ray beam quality is essential in diagnostic radiography for patient-dose optimization, image-quality assurance, and equipment quality control (QC). The half-value layer (HVL), defined as the thickness of an attenuating material required to reduce the incident air kerma to one half, is widely used as a practical index of X-ray beam hardness [1,2]. Semiconductor-based solid-state X-ray meters are increasingly used in routine QC because they provide rapid readout and convenient handling [3]. Conventional HVL determination commonly relies on sequential aluminum attenuation measurements using ionization-chamber dosimetry systems [4,5]. Under ideal narrow-beam conditions, HVL should be largely independent of source-to-detector distance because geometric inverse-square effects are common to both attenuated and unattenuated measurements [6].

Previous studies have evaluated the accuracy and applicability of solid-state detectors and non-invasive X-ray analyzers for diagnostic X-ray beam-quality assessment [7-11]. Ioka et al. compared a non-invasive X-ray output analyzer with reference measurement instruments under conventional radiographic conditions and reported good agreement for several measured parameters, including HVL [9]. Maruyama et al. evaluated the measurement accuracy and uncertainty of a solid-state detector under diagnostic X-ray beam conditions and demonstrated that detector response may vary depending on beam quality and measurement conditions [7]. Lin and Goode also evaluated the accuracy of solid-state detectors for HVL measurements in radiography and fluoroscopy systems [8]. More recently, Stasko and Culberson investigated the performance and long-term stability of X-ray multimeters under calibration-laboratory conditions, including HVL measurements, and emphasized the importance of calibration stability and periodic verification [10]. Murata et al. further demonstrated the feasibility of using a solid-state detector-based system to evaluate general X-ray equipment parameters, including HVL, in a rapid measurement procedure [11].

Cobia SMART (RTI Group, Mölndal, SWE) directly reports HVL together with other X-ray output parameters, making it attractive for rapid equipment checks. However, although previous investigations generally support the usefulness of solid-state and non-invasive measurement systems, agreement with conventional ionization-chamber measurements may depend on beam quality, detector characteristics, calibration, irradiation geometry, and exposure conditions [7-11]. Therefore, agreement demonstrated under a limited number of conditions should not automatically be generalized across the entire diagnostic radiography range.

In practical radiographic measurements, scattered radiation, field geometry, detector positioning, exposure level, detector energy response, and the interpolation procedure may introduce measurable deviations. Therefore, this study compared first-HVL measurements obtained using Cobia SMART and an ionization-chamber dosimetry system at four tube voltages (60, 80, 100, and 120 kV) and three source-to-detector distances (100, 150, and 200 cm). The objectives were to evaluate inter-device agreement, measurement repeatability, and variations observed under different tube-voltage and measurement-distance conditions using the complete experimental dataset.

## Materials and methods

Study design and equipment

This benchtop experimental study involved no human participants, patient data, biological specimens, or animals. Institutional review board approval and informed consent were therefore not applicable. Measurements were performed using a Shimadzu UD150L-40 general radiography X-ray system (Shimadzu Corp., Kyoto, JPN). A Cobia SMART R/F (RTI Group, Mölndal, SWE) was used as the solid-state X-ray meter. Reference measurements were obtained using a Radcal Model 9015 dosimetry system (Radcal Corp., Monrovia, CA, USA) equipped with an ionization chamber. The experimental records indicated that the measurement instruments had been calibrated; however, numerical calibration-certificate uncertainty values were not available and were therefore not assigned retrospectively.

Exposure conditions and measurement geometry

Measurements were performed at tube voltages of 60, 80, 100, and 120 kV and source-to-detector distances (SDDs) of 100, 150, and 200 cm. The corresponding exposure settings were 50, 110, and 200 mAs, with exposure times of 200, 280, and 500 ms, respectively. The mAs was increased with increasing SDD to approximately compensate for the inverse-square reduction in detector exposure and to maintain an adequate measurement signal. The selected mAs values approximately scaled with the square of SDD, consistent with compensation for the inverse-square reduction in detector exposure. Accordingly, mAs was not held constant across the three distance conditions. Because mAs and SDD were changed simultaneously, the experiment was not designed to evaluate SDD as an isolated independent variable.

The detector was positioned on the central X-ray beam axis, and the irradiation field was collimated to a small area intended to approximate near-narrow-beam geometry and minimize scattered radiation. The measurement setup followed general diagnostic dosimetry principles for beam-quality assessment [12,13]. However, formal compliance with a specific International Electrotechnical Commission (IEC) 61267 radiation quality was not independently verified.

Ionization-chamber HVL determination

For each tube-voltage and SDD condition, the unattenuated ionization-chamber signal was first measured. Aluminum (Al) attenuators were then added sequentially in nominal 1-mm increments to achieve a range of total Al thicknesses. At each Al thickness, three signal readings were obtained and averaged. Transmission was normalized to the corresponding mean unattenuated signal. The first HVL was determined from the two adjacent Al thicknesses that bracketed 50% transmission using logarithmic interpolation: \(\text{HVL} = x_1 + (x_2 - x_1) \times \frac{\ln(T_1 / 0.5)}{\ln(T_1 / T_2)}\, where T₁ > 0.5 and T₂ < 0.5 are the normalized transmission values at the corresponding Al thicknesses x₁ and x₂, respectively. Three HVL determinations were obtained for each tube-voltage/SDD combination and were used to assess measurement repeatability.

Cobia HVL measurement

Cobia SMART provides a direct HVL estimate based on its internal detector response and processing algorithm. Three Cobia HVL readings were recorded for each tube-voltage/SDD condition. All Cobia HVL values were recorded to one decimal place, corresponding to an increment of 0.1 mm Al. Therefore, identical triplicate recorded values were interpreted as indicating no detectable variation at the recorded resolution rather than zero underlying measurement uncertainty.

Repeatability and agreement analysis

For the ionization-chamber HVL measurements, the arithmetic mean, sample SD, and coefficient of variation (CV) were calculated from the three determinations obtained under each experimental condition. The CV was calculated as: $\text{CV } (\%) = \frac{\mathrm{SD}}{\mathrm{mean}} \times 100$. For each of the 12 paired experimental conditions, the signed inter-device difference was defined as the Cobia HVL value minus the mean ionization-chamber HVL value. The percentage difference was calculated relative to the mean ionization-chamber HVL value. The mean absolute percentage difference was also calculated across all 12 conditions.

Agreement between the two measurement methods was further explored using Bland-Altman analysis [14] based on the 12 paired condition means. The mean paired difference was defined as the bias, and the descriptive 95% limits of agreement (LoA) were calculated as $\mathrm{LoA} = \mathrm{bias} \pm 1.96 \times \mathrm{SD}$ of the paired differences. Because the dataset consisted of only 12 predefined engineering test conditions obtained from a single X-ray system rather than a random sample of independent clinical observations, the Bland-Altman statistics were interpreted descriptively. No hypothesis testing or population-level claim of equivalence was made. The analysis was intended to provide a preliminary, condition-specific assessment of agreement between the two measurement systems.

Uncertainty assessment

Measurement uncertainty was evaluated according to the general framework of the Guide to the Expression of Uncertainty in Measurement (GUM) [15]. For the ionization-chamber method, the repeatability-based type A standard uncertainty of the condition mean was calculated as $u{\mathrm{A,ion}} = \frac{\mathrm{SD}}{\surd n{}}$, where SD is the sample standard deviation of the repeated HVL determinations and n is the number of repeated determinations. Because three determinations were obtained for each experimental condition, n = 3; therefore: $u{\mathrm{A,ion}} = \frac{\mathrm{SD}}{\surd3{}}$.

For Cobia SMART, HVL values were recorded in increments of 0.1 mm Al. Assuming rounding to the nearest 0.1 mm Al, the possible rounding interval was ±0.05 mm Al. Assuming a rectangular distribution within this interval, the corresponding recording-resolution standard uncertainty was calculated as $u{\mathrm{rec,Cobia}} = \frac{0.05}{\sqrt{3}} = \frac{0.1}{\sqrt{12}} = 0.0289\text{ mm Al}$. Assuming that the ionization-chamber repeatability component and the Cobia recording-resolution component were independent, the partial combined standard uncertainty of the paired difference was calculated using the root-sum-of-squares method: $u{\mathrm{partial}} = \surd{u{\mathrm{A,ion}}^2 + u{\mathrm{rec,Cobia}}^2}$. The corresponding partial expanded uncertainty was calculated using a coverage factor of k = 2: $U{\mathrm{partial}} = 2 \times u{\mathrm{partial}}$.

This uncertainty assessment intentionally included only components that could be quantified from the available experimental records. A complete metrological uncertainty budget would additionally require calibration-certificate uncertainties for both measurement systems, ionization-chamber energy dependence, Cobia spectral-response and correction uncertainty, Al-filter thickness and purity tolerances, tube-voltage reproducibility, detector positioning, irradiation-field effects, scattered radiation, and interpolation/model uncertainty. Because these Type B uncertainty components were unavailable, no complete combined or expanded uncertainty was claimed, and the partial uncertainty estimates were not used to establish formal metrological equivalence.

## Results

Condition-specific HVL values and repeatability

Half-value layer measurements were obtained under 12 predefined combinations of tube voltage and SDD. The condition-specific results are summarized in Table 1. For the ionization-chamber method, the CV ranged from 0.09% to 2.38%, with a mean CV of 1.16% across all conditions. The smallest CV was observed at 100 kV and 200 cm, whereas the largest CV was observed at 120 kV and 100 cm. Cobia SMART produced identical recorded triplicate HVL values at all three SDDs for each tube voltage. The recorded values were 2.1 mm Al at 60 kV, 2.9 mm Al at 80 kV, 3.7 mm Al at 100 kV, and 4.6 mm Al at 120 kV. Because Cobia values were recorded to one decimal place, these identical triplicate values indicate no detectable variation at the recorded resolution rather than zero underlying measurement variability.

**Table 1:** HVL measurements by tube voltage and source-to-detector distance Ionization-chamber values represent the mean of three independent HVL determinations. The difference was calculated as the Cobia HVL value minus the mean ionization-chamber HVL value. The percentage difference was calculated relative to the mean ionization-chamber HVL value. HVL: Half-value layer; SDD: Source-to-detector distance; SD: Standard deviation; CV: Coefficient of variation; Al: Aluminum

kV	SDD (cm)	Ion mean (mm Al)	SD	CV (%)	Cobia (mm Al)	Difference (mm Al)	Difference (%)
60	100	2.088	0.035	1.65	2.1	0.012	0.55
60	150	2.126	0.020	0.96	2.1	-0.026	-1.21
60	200	2.197	0.021	0.96	2.1	-0.097	-4.41
80	100	2.954	0.052	1.75	2.9	-0.054	-1.83
80	150	2.778	0.027	0.98	2.9	0.122	4.40
80	200	2.887	0.007	0.25	2.9	0.013	0.44
100	100	3.727	0.057	1.54	3.7	-0.027	-0.72
100	150	3.526	0.015	0.42	3.7	0.174	4.93
100	200	3.650	0.003	0.09	3.7	0.050	1.38
120	100	4.651	0.111	2.38	4.6	-0.051	-1.09
120	150	4.134	0.086	2.08	4.6	0.466	11.27
120	200	4.504	0.038	0.85	4.6	0.096	2.14

Inter-device agreement

Across the 12 predefined experimental conditions, the mean absolute percentage difference between Cobia SMART and the ionization-chamber method was 2.86%. In 11 of the 12 conditions, the absolute difference between the Cobia HVL value and the mean ionization-chamber HVL value was no greater than 0.20 mm Al. The largest discrepancy occurred at 120 kV and 150 cm. Under this condition, the mean ionization-chamber HVL was 4.134 mm Al, whereas the Cobia value was 4.6 mm Al, corresponding to a difference of +0.466 mm Al and a percentage difference of +11.27%.

A descriptive Bland-Altman analysis of the 12 paired condition means yielded a mean bias of +0.057 mm Al, indicating that Cobia values were slightly higher on average. The descriptive 95% limits of agreement ranged from -0.240 to +0.353 mm Al. The 120 kV/150 cm condition exceeded the upper descriptive limit of agreement and contributed substantially to the width of the overall limits (Figure 1). Because the analysis was based on only 12 predefined engineering test conditions from a single X-ray system, these Bland-Altman results were interpreted descriptively and were not used to establish statistical or metrological equivalence between the two measurement systems.

**Figure 1:**
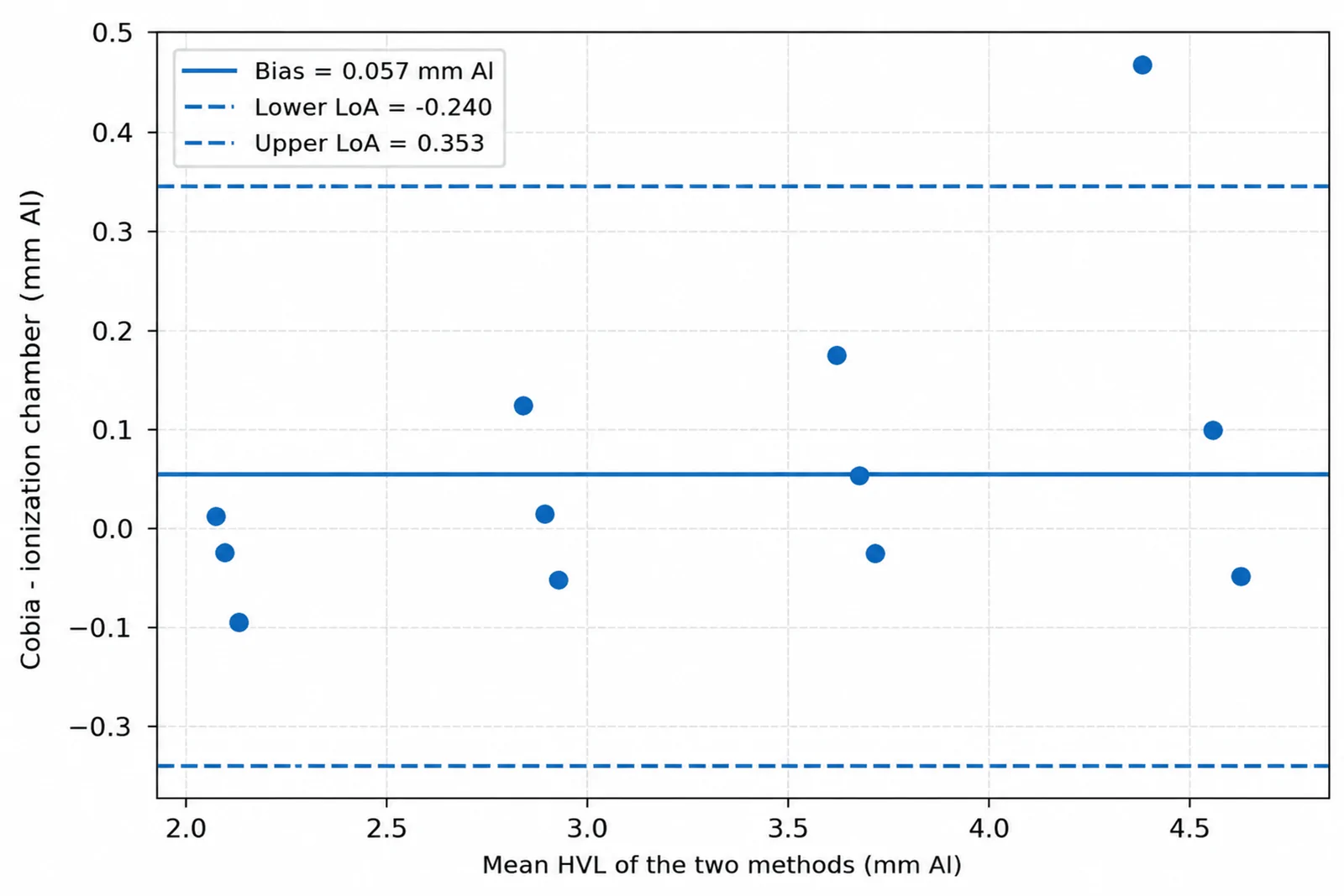
Bland-Altman plot Bland-Altman plot of the 12 paired condition means. Differences are calculated as Cobia minus the ionization chamber. The solid line represents the mean bias of +0.057 mm Al, and the dashed lines represent the descriptive 95% limits of agreement from -0.240 to +0.353 mm Al. The limits are interpreted descriptively because of the small engineering dataset. Al: Aluminum

Uncertainty-related results

The repeatability-based type A standard uncertainty of the ionization-chamber condition mean, calculated as SD/√3, ranged from 0.0019 to 0.0638 mm Al. The recording-resolution standard uncertainty assigned to the Cobia data based on the 0.1-mm Al recording increment was 0.0289 mm Al. Combining the ionization-chamber repeatability component and the Cobia recording-resolution component yielded a partial combined standard uncertainty ranging from 0.0289 to 0.0700 mm Al.

The corresponding partial expanded uncertainty using a coverage factor of k = 2 ranged from 0.0579 to 0.1401 mm Al. These values represent incomplete uncertainty estimates because the unavailable type B uncertainty components were not included. The uncertainty components supported by the available dataset are summarized in Table 2.

**Table 2:** Uncertainty components supported by the supplied dataset The reported partial uncertainties include only components that could be quantified from the available experimental records and do not represent a complete metrological uncertainty budget. HVL: Half-value layer; GUM: Guide to the expression of uncertainty in measurement; Al: Aluminum; uA,ion: Repeatability-based type A standard uncertainty of the ionization-chamber mean; urec,Cobia: Standard uncertainty associated with the Cobia recording increment

Component	Evaluation	Standard uncertainty	Interpretation
Ion-chamber repeatability	Type A: SD/√3	0.0019-0.0638 mm Al	Condition-specific; n = 3 HVL determinations
Cobia worksheet recording increment	Type B: rectangular, 0.1/√12	0.0289 mm Al	Based on recorded 0.1-mm Al increment, not asserted as manufacturer specification
Partial paired standard uncertainty	√(uA,ion² + urec,Cobia²)	0.0289-0.0700 mm Al	Repeatability + recording increment only
Partial expanded uncertainty	k = 2	0.0579-0.1401 mm Al	Not a complete uncertainty budget
Calibration, energy response, Al filters, geometry/scatter	Type B	Not quantified	Required for a complete GUM uncertainty budget

Tube-voltage and measurement-distance patterns

The HVL increased with increasing tube voltage for both measurement systems. The mean ionization-chamber HVL values were not completely invariant across the three measurement-distance conditions. The across-distance spread was 0.108 mm Al at 60 kV, 0.176 mm Al at 80 kV, 0.201 mm Al at 100 kV, and 0.516 mm Al at 120 kV.

Cobia SMART recorded the same one-decimal HVL value at all three distances for each tube voltage. Because mAs was intentionally increased with SDD to approximately compensate for the inverse-square reduction in detector exposure, SDD and mAs were changed simultaneously. Therefore, these results represent combined measurement-distance and exposure conditions and should not be interpreted as an isolated assessment of SDD dependence. The relationships between tube voltage and HVL at SDDs of 100, 150, and 200 cm are shown in Figures 2-4, respectively.

**Figure 2:**
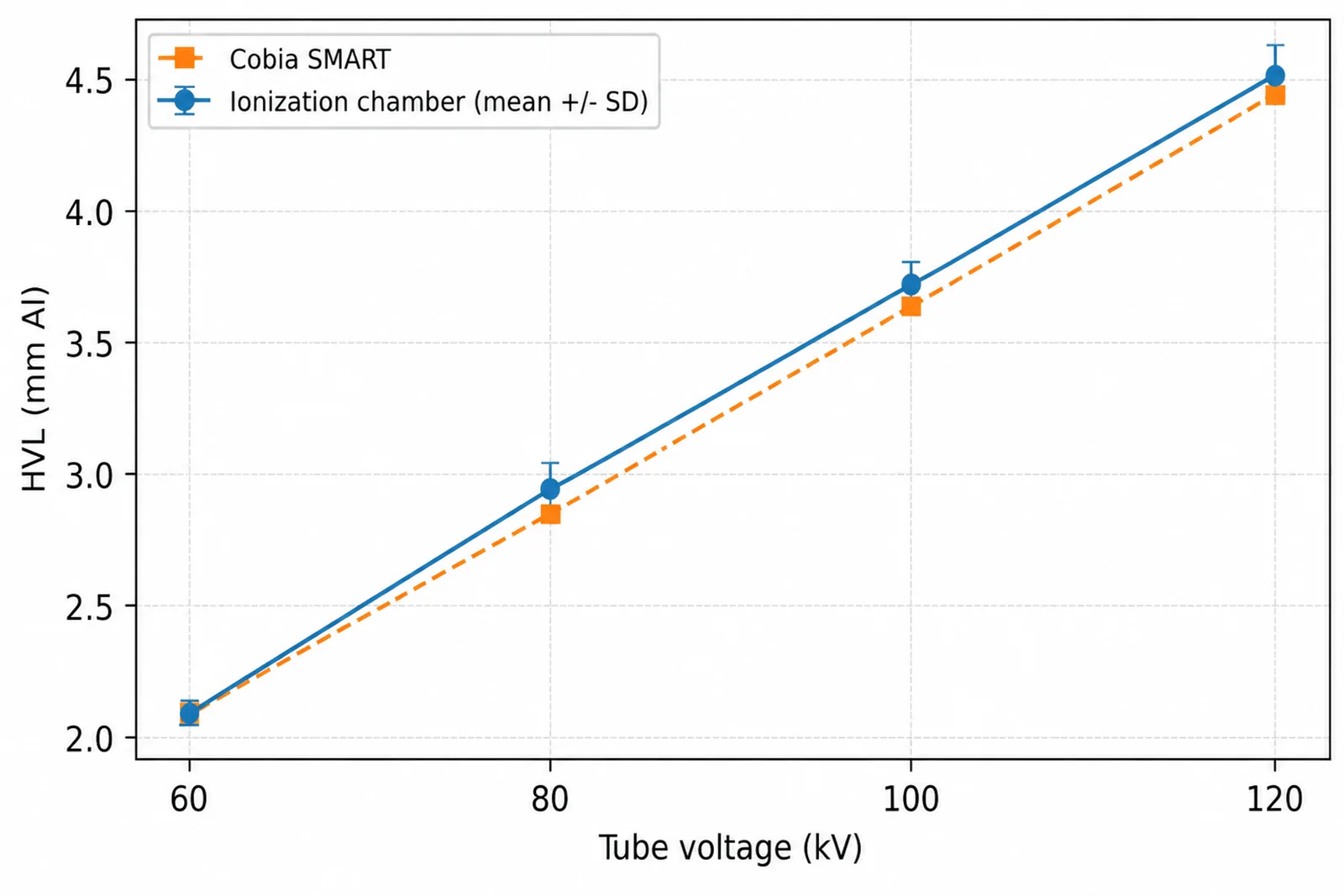
HVL and tube voltage comparison at an SDD of 100 cm Ionization-chamber values are means with SD error bars; Cobia values are recorded as direct HVL readings. HVL: Half-value layer; SDD: Source-to-detector distance

**Figure 3:**
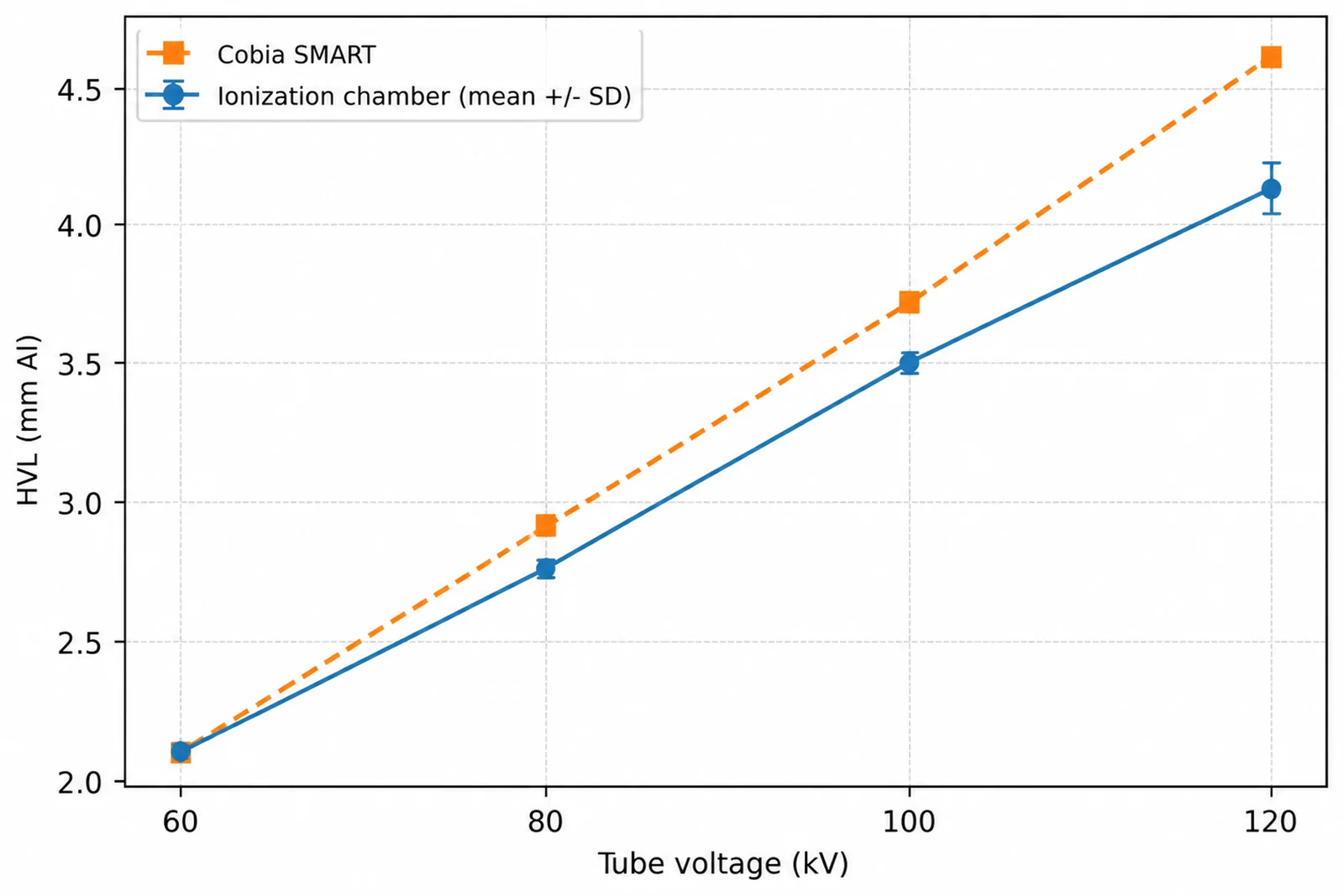
HVL and tube voltage comparison at an SDD of 150 cm Ionization-chamber values are means with SD error bars; Cobia values are recorded as direct HVL readings. HVL: Half-value layer; SDD: Source-to-detector distance

**Figure 4:**
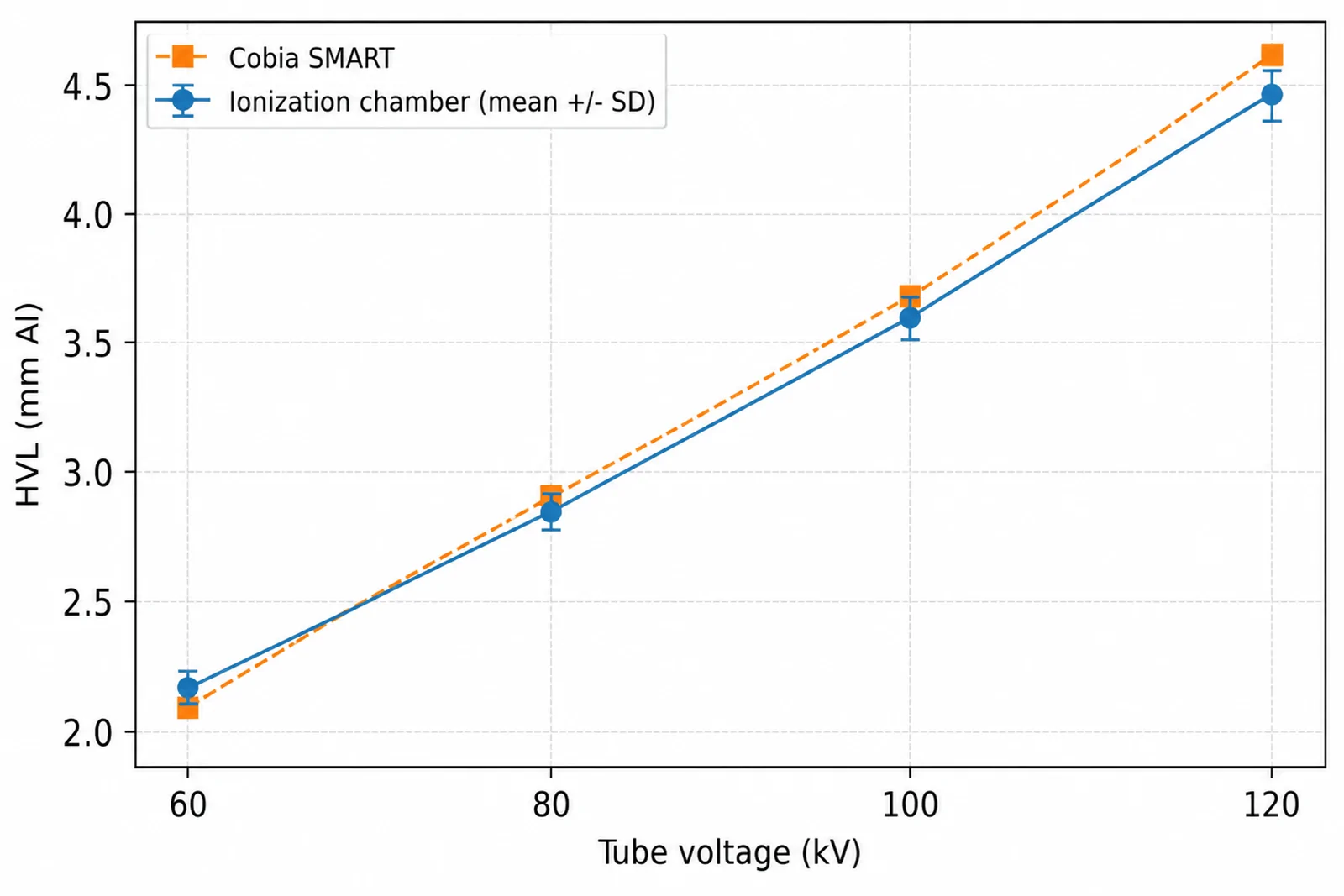
HVL and tube voltage comparison at an SDD of 200 cm Ionization-chamber values are means with SD error bars; Cobia values are recorded as direct HVL readings. HVL: Half-value layer; SDD: Source-to-detector distance

## Discussion

The present study compared HVL measurements obtained using Cobia SMART and an ionization-chamber dosimetry system across four tube voltages and three measurement-distance conditions. Overall, the two methods showed close agreement under most of the predefined experimental conditions. Eleven of the 12 paired condition means differed by no more than 0.20 mm Al, and the mean absolute percentage difference was 2.86%. However, a notable discrepancy was observed at 120 kV and 150 cm. Because the study comprised only 12 predefined engineering test conditions from a single X-ray system, these findings should be interpreted as a preliminary, condition-specific comparison rather than as a definitive demonstration of equivalence between the two measurement systems.

Ionization chambers have traditionally been used for diagnostic X-ray beam-quality measurements because of their established dosimetric characteristics, whereas solid-state detectors and non-invasive X-ray analyzers offer practical advantages including compact size, rapid readout, and operational simplicity [3,9-11]. Previous investigations have generally demonstrated useful agreement between solid-state systems and reference measurements, although the magnitude of disagreement may depend on beam quality, detector characteristics, calibration, and measurement conditions [7-10]. Ioka et al. compared a non-invasive X-ray output analyzer with reference instruments under conventional radiographic conditions and reported HVL differences within approximately ±4.3% [9]. Murata et al. similarly demonstrated the feasibility of a solid-state detector-based system for rapid evaluation of X-ray equipment parameters, including HVL [11]. More recently, Stasko and Culberson evaluated X-ray multimeter performance under calibration-laboratory conditions and reported generally satisfactory HVL performance while emphasizing the importance of calibration stability and periodic verification [10].

The mean absolute percentage difference of 2.86% observed in the present study is broadly consistent with these previous reports. Nevertheless, the +11.27% discrepancy observed at 120 kV and 150 cm indicates that close agreement under most tested conditions should not be interpreted as universal interchangeability between Cobia SMART and the ionization-chamber method. Importantly, the 120 kV/150 cm condition was not independently repeated in a separate confirmation experiment. The observed discrepancy should therefore be regarded as a condition-specific observation requiring confirmation rather than evidence of a systematic high-kV measurement bias.

The ionization-chamber measurements demonstrated good short-term repeatability. Condition-specific coefficients of variation ranged from 0.09% to 2.38%, with a mean CV of 1.16%. These findings indicate that large random variation in the repeated ionization-chamber measurements alone is unlikely to explain all of the observed inter-device differences. Cobia SMART produced identical recorded triplicate values for each tube voltage at all three distances. However, the Cobia values available for this study were recorded to one decimal place. Therefore, identical recorded values should not be interpreted as evidence of zero underlying measurement uncertainty; rather, no variation was detectable within the recorded 0.1-mm Al increment.

The present findings also highlight the importance of measurement conditions when comparing solid-state and reference dosimetry systems. Previous studies have shown that solid-state detector response may vary with beam quality, spectral characteristics, and measurement geometry [7,8]. Long-term calibration stability and detector-specific response characteristics may also influence agreement between X-ray multimeters and reference systems [10]. These considerations are relevant to the present dataset because the largest inter-device discrepancy occurred at the highest tube voltage investigated, although the other two 120-kV conditions showed substantially smaller differences. Therefore, the data do not support a general conclusion that Cobia SMART systematically overestimates HVL at high tube voltage.

Under ideal narrow-beam conditions, HVL is expected to be largely independent of SDD distance because geometric inverse-square effects influence both attenuated and unattenuated measurements and therefore largely cancel when transmission ratios are calculated [4,6]. In the present experiment, however, mAs was intentionally increased as SDD increased to approximately compensate for the inverse-square reduction in detector exposure and to maintain an adequate measurement signal. The selected mAs values approximately scaled with the square of SDD. Consequently, SDD and mAs were changed simultaneously, and the experiment was not designed to assess SDD as an isolated independent variable.

The ionization-chamber HVL values nevertheless showed some variation among the three distance/exposure conditions, with across-condition spreads of 0.108 mm Al at 60 kV, 0.176 mm Al at 80 kV, 0.201 mm Al at 100 kV, and 0.516 mm Al at 120 kV. Several factors may have contributed to these differences, including residual scattered radiation, detector positioning, irradiation-field geometry, generator output characteristics, interpolation sensitivity, and detector-specific energy response [4,7,8]. The present experiment was not designed to distinguish among these potential mechanisms. Additional measurements with mAs held constant and repeat measurements of the 120 kV/150 cm condition would be useful in a future confirmatory study.

The Bland-Altman analysis was included as a descriptive assessment of agreement across the 12 predefined condition means. The mean bias was +0.057 mm Al, indicating only a small overall tendency for Cobia SMART to produce higher recorded values than the ionization-chamber method. The descriptive 95% limits of agreement ranged from -0.240 to +0.353 mm Al, and the 120 kV/150 cm condition exceeded the upper limit. This condition materially widened the overall limits. Because the analysis was based on only 12 engineering test conditions from one X-ray system rather than a random sample of independent clinical observations, the limits of agreement should not be interpreted as precise population estimates or as evidence of statistical equivalence.

The uncertainty analysis was likewise intentionally limited to components that could be quantified from the available experimental records. Repeatability-based type A uncertainty for the ionization-chamber measurements and a recording-resolution component for Cobia SMART were included according to the general GUM framework [15]. The resulting partial expanded uncertainty ranged from 0.0579 to 0.1401 mm Al. These values represent partial engineering estimates rather than a complete uncertainty budget because calibration-certificate uncertainty, detector energy dependence, aluminum-filter thickness and purity, positioning, scatter, and interpolation/model uncertainty were unavailable. The +0.466-mm Al difference observed at 120 kV and 150 cm was larger than the partial expanded uncertainty derived from the available components. However, because the complete type B uncertainty budget was unavailable, formal metrological incompatibility between the two methods cannot be established from the present data.

From a routine quality-control perspective, Cobia SMART remains attractive because it provides rapid direct HVL measurements without the sequential attenuation procedure required for conventional ionization-chamber determination. This practical advantage is consistent with previous reports concerning non-invasive and solid-state measurement systems for diagnostic X-ray quality assurance [9-11]. The close agreement observed in 11 of the 12 predefined conditions suggests the potential usefulness of Cobia SMART for rapid routine surveillance and educational QC measurements. However, the limited dataset and the single notable discrepancy indicate that periodic comparison with a calibrated reference dosimetry system remains advisable, particularly at higher tube voltages, after changes in measurement geometry, following instrument recalibration, or when unexpected HVL values are obtained.

Limitations

Several limitations should be considered when interpreting these findings. First, only one general radiography system and one model of each measurement system were evaluated; therefore, the results cannot be generalized to all X-ray generators or solid-state meters. Second, the study included only 12 predefined paired experimental conditions, with three repeated HVL determinations per condition. This limited dataset is insufficient for strong population-level statistical inference, and the Bland-Altman analysis should therefore be considered descriptive and exploratory. Third, the discrepancy observed at 120 kV and 150 cm was not independently repeated in a separate confirmation experiment. This finding requires replication before any conclusion regarding a condition-specific or high-kV measurement bias can be made. Fourth, mAs was intentionally increased simultaneously with SDD to approximately compensate for the inverse-square reduction in detector exposure. Consequently, SDD was not evaluated as an isolated independent variable, and the observed differences among distance conditions cannot be attributed solely to distance. Fifth, Cobia SMART results were recorded to one decimal place in the experimental worksheet, which may have concealed small within-condition variations. Sixth, numerical calibration-certificate uncertainties, the ionization-chamber head model and active volume, Al-filter purity and thickness tolerances, and quantitative assessments of scatter and positioning uncertainty were unavailable. A complete GUM-based uncertainty budget could therefore not be constructed. Seventh, the experimental geometry was intended to approximate near-narrow-beam conditions, but formal verification against a specified IEC 61267 radiation quality was not performed. Finally, only the first HVL was evaluated. Future studies should include a larger number of measurement conditions, repeated confirmation of discrepant conditions, measurements with mAs held constant when evaluating SDD effects, and, where feasible, evaluation of the second HVL, homogeneity coefficient, and direct spectral characteristics.

## Conclusions

Within this limited set of 12 predefined engineering conditions, Cobia SMART showed generally close agreement with the ionization-chamber method for first half-value layer (HVL) measurements. Eleven of the 12 paired condition means differed by no more than 0.20 mm Al, and the mean absolute percentage difference was 2.86%. Ionization-chamber repeatability was good, with coefficients of variation ranging from 0.09% to 2.38%. However, a notable discrepancy of +0.466 mm Al (+11.27%) was observed at 120 kV and 150 cm, and the descriptive Bland-Altman limits of agreement ranged from -0.240 to +0.353 mm Al.

Because the study was based on a small number of predefined conditions from a single X-ray system, mAs and SDD were changed simultaneously, and a complete type B uncertainty budget was unavailable, the findings should be regarded as preliminary and condition-specific rather than as evidence of universal metrological equivalence. Cobia SMART nevertheless appears potentially useful for rapid routine and educational HVL quality-control measurements. Further studies using larger measurement series, repeated assessment of discrepant conditions, and measurements with mAs held constant when evaluating SDD effects are warranted.
